# De-regulation of common housekeeping genes in hepatocellular carcinoma

**DOI:** 10.1186/1471-2164-8-243

**Published:** 2007-07-18

**Authors:** Samuel Waxman, Elisa Wurmbach

**Affiliations:** 1 Mount Sinai School of Medicine, Department of Medicine, Division of Hematology/Oncology, New York, NY, USA

## Abstract

**Background:**

Tumorigenesis is associated with changes in gene expression and involves many pathways. Dysregulated genes include "housekeeping" genes that are often used for normalization for quantitative real-time RT-PCR (qPCR), which may lead to unreliable results. This study assessed eight stages of hepatitis C virus (HCV) induced hepatocellular carcinoma (HCC) to search for appropriate genes for normalization.

**Results:**

Gene expression profiles using microarrays revealed differential expression of most "housekeeping" genes during the course of HCV-HCC, including glyceraldehyde-3-phosphate dehydrogenase (GAPDH) and beta-actin (ACTB), genes frequently used for normalization. QPCR reactions confirmed the regulation of these genes. Using them for normalization had strong effects on the extent of differential expressed genes, leading to misinterpretation of the results.

**Conclusion:**

As shown here in the case of HCV-induced HCC, the most constantly expressed gene is the arginine/serine-rich splicing factor 4 (SFRS4). The utilization of at least two genes for normalization is robust and advantageous, because they can compensate for slight differences of their expression when not co-regulated. The combination of ribosomal protein large 41 (RPL41) and SFRS4 used for normalization led to very similar results as SFRS4 alone and is a very good choice for reference in this disease as shown on four differentially expressed genes.

## Background

Cancer development affects almost all pathways and genes [1-4]. Also affected are the so-called "housekeeping" genes, which are involved in the cell's common basic functions [5-8]. Typical housekeeping genes include glyceraldehyde-3-phosphate dehydrogenase (GAPDH), beta-actin (ACTB), TATA-binding protein (TBP), ribosomal proteins (RP), and many more [9-14]. Many of these genes are often used to normalize quantitative real-time RT-PCR (qPCR) data [13,15,16] to account for experimental differences, such as differences in RNA quantity and quality, the overall transcriptional activity and differences in the cDNA synthesis. GAPDH and ACTB are most commonly used for normalization [17-21], including studies on cancer [22-24]. Despite the fact that it was shown that these genes are differentially expressed in cancers, including colorectal-, prostate- and bladder-cancer. [6-8,25]. Some qPCR studies on hepatocellular carcinoma (HCC) used GAPDH or ACTB for normalization [26-28].

Many investigations on cancer include multiple comparisons, by analyzing different stages of the disease, such as normal tissue, pre-neoplasm, and consecutive stages of cancer [29-32]. Such an experimental design makes it crucial to find an appropriate gene for normalization. Prerequisites for normalization genes are constant expression throughout all disease stages and no response to treatment. Extensive evidence indicates that all genes can be regulated under some conditions.

This study focuses on hepatitis C virus (HCV) induced hepatocellular carcinoma (HCC), comprising eight pathological stages, including pre-neoplastic lesions (cirrhosis and dysplasia) and four consecutive stages of HCC and reveals that many of the 'housekeeping" genes are indeed differentially expressed. In addition, the effects of different reference genes used for normalization on differentially expressed genes are presented and appropriate genes useful for normalization when investigating HCV-induced HCC are introduced.

## Results

### Typical "housekeeping" genes are deregulated in HCV-induced HCC

**Figure 1:**
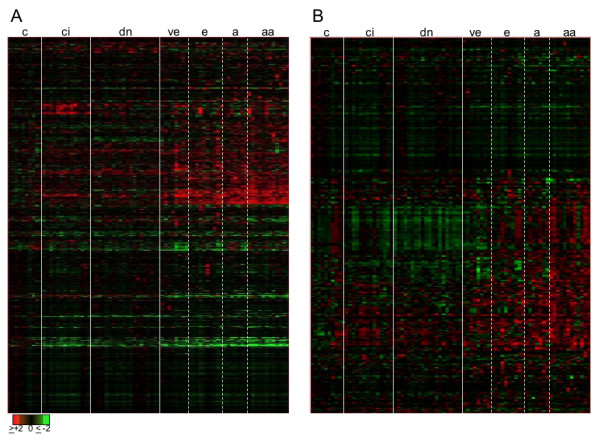
**Common "housekeeping" genes are deregulated in HCV-induced HCC (multiple comparison microarray data)**. A) Gene expression of over 30,000 genes that showed no change to controls in at least one of 72 samples studied. B) 323 common "housekeeping" genes whose products have functions in sugar-, nucleotide-, lipid-, amino acid-, and energy-metabolism, or code for ribosomal proteins, basal transcription factors, and proteins of the cytoskeleton. In A) and B) the columns correspond to the stages of the disease: c = control, ci = cirrhosis, dn = dysplasia, ve = very early HCC, e = early HCC, a = advanced HCC, and aa = very advanced HCC. Genes (in rows) were clustered using the Pearson correlation. Red indicates up-regulation, green down-regulation, and black no change or not expressed.

Analyzing the expression profile of all stages of HCV-induced HCC, including preneoplastic stages (cirrhosis and dysplasia) and four cancerous stages with microarrays revealed that almost all pathways were affected [4]. In order to find normalization genes for qPCR verification, we looked for genes that showed no differential expression in any of the eight stages analyzed. First, we selected genes that displayed no change to controls in at least one sample of the 72 samples included. This resulted in a list of over 30,000 genes (Figure 1A). Among these, many genes showed an increased expression in cancerous stages compared to normal liver controls or were not expressed in the liver and tumor tissues (absent call). In addition, some genes were down-regulated in certain stages of the disease. Hence, most of these genes were inappropriate to be used as reference gene for normalization. In further selection steps, we thus excluded genes that were regulated or that were not expressed (absent call) in any of the stages of the disease. This procedure led to a list of 46 genes, including 27 genes coding for ribosomal proteins and five genes coding for splicing factors. Thus, excluding differentially expressed genes led to only few genes that were expressed in all stages and not changed during the course of HCV-induced HCC: The best candidates for normalization were RPL41 and SFRS4. Genes of different pathways were chosen to exclude the possibility of co-regulation.

Furthermore, specifically checking housekeeping genes, with functions in sugar-, nucleotide-, lipid-, amino acid-, or energy-metabolism, or ribosomal proteins, basal transcription factors and proteins of the cytoskeleton (Figure 1B), we found that most of them were either differentially expressed during disease progression or not expressed at all. These results display clearly that housekeeping genes are affected in HCV-induced HCC.

### Candidate reference genes from multiple comparison microarray data

In a different approach to identify genes appropriate for normalization from a microarray study comprising multiple comparisons we calculated the standard deviation (SD) of all fold changes for each gene. Genes with a low SD across all fold-changes and similar signal intensities to the genes of interest (or present call) may provide a pool of normalization candidates, for qPCR (see below).

Six genes were chosen as candidate reference genes for the purpose of this study: RPL41 and SFRS4 and the commonly used reference genes GAPDH, ACTB and TBP, as well as another gene coding for a ribosomal protein, RPS20. The SD of their fold changes (microarray data) ranks them as follows: RPL41 (0.09), ACTB (0.23), SFRS4 (0.24), TBP (0.28), GAPDH (0.34), and lastly RPS20 (0.43).

### Reference genes for HCV-induced HCC

**Figure 2:**
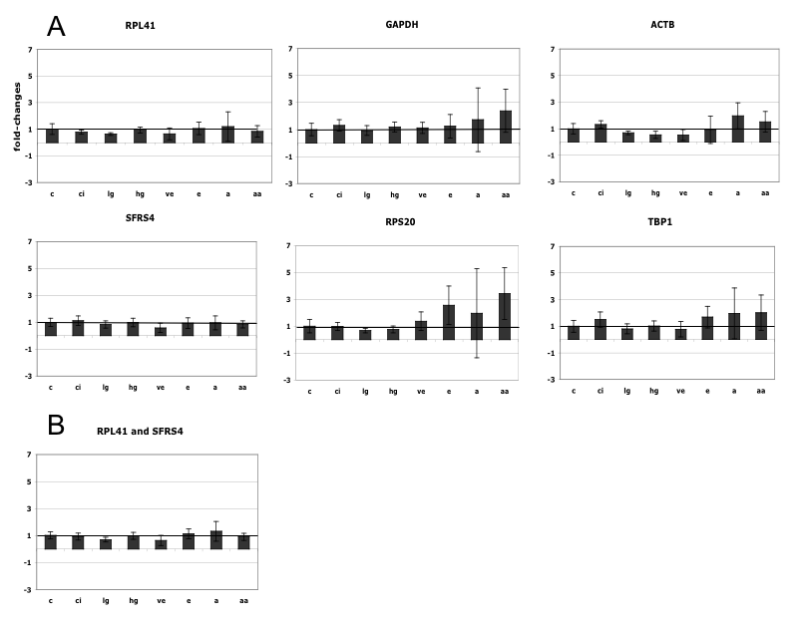
**QPCR: expression of candidate genes for normalization of HCV-induced HCC**. Plotted are median fold-changes (relative quantification with respect to the median Ct of the control samples, corrected for PCR-efficiencies) plus minus SD for each stage of the disease: c = control (n = 10), ci = cirrhosis (n = 10), lg = low-grade dysplasia (n = 10), hg = high-grade dysplasia (n = 7), ve = very early HCC (n = 8), e = early HCC (n = 10), a = advanced HCC (n = 7), and aa = very advanced HCC (n = 10). A) Expression of RPL41, GAPDH, ACTB, SFRS4, RPS20, and TBP. B) Average of the expression of RPL41 and SFRS4.

Quantitative real-time PCR (qPCR) was performed for RPL41, SFRS4, GAPDH, ACTB, RPS20, and TBP on all tissue samples. These qPCRs were performed twice (each in triplicate), to reduce the technical variation. First, we compared the SD of their Ct values (n = 72), which was lowest for SFRS4 (0.63), followed by RPL41 (0.82), GAPDH (0.91), TBP (1.03), ACTB (1.07), and RPS20 (1.19). For each reaction, we calculated relative expression levels, by subtracting the median Ct of control samples from all other Ct values, followed by determining (1+E)^-ΔCt ^(see Methods). Figure 2 shows these data for the six candidate genes for each stage of HCV-induced HCC. Notably, the variation increases at later disease stages (early to very advanced HCC). The coefficient of variation (CV) allows comparison of the variation of gene-expressions independent of their mean value. The CV was smallest for SFRS4 (38%), followed by RPL41 (53%), ACTB (65%), TBP (70%), GAPDH (75%), and RPS20 (94%).

Importantly, GAPDH was significantly up-regulated in advanced stages of HCC, as calculated by the Student's t-test (p = 0.016 control vs. very advanced HCC). Even more obvious was the up-regulation of RPS20 during HCC, which was already significant between control and early HCC (p = 0.003). TBP and ACTB also showed a significant up-regulation between control and very advanced HCC (p = 0.014, p = 0.011, respectively).

We also used the geNorm program [13], to determine the best normalization gene for HCV-induced HCC by stepwise exclusion of the least stable expressed gene. The most stably expressed genes were RPL41 and SFRS4, resulting in M = 0.65, M describing the average expression stability (lowest for the most stably expressed genes). The expression stabilities for TBP (M = 0.74), ACTB (M = 0.78), GAPDH (M = 0.82), and RPS20 (M = 0.88) were worse. Hence, again, RPL41 and SFRS4 (Figure 2B) were the best candidates for normalization of HCV-induced HCC.

### Effects of different genes used for normalization

Normalization is used to adjust for experimental differences. In qPCR normalization corrects for the RNA quantity, the overall transcriptional activity, the cDNA synthesis and the PCR efficiency. Ideally, a reference gene is an internal endogenous control, shows constant expression in the tissue under investigation and does not respond to the experimental treatment.

Four commonly used "housekeeping" genes (GAPDH, ACTB, RPS20, TBP1) and the combined data of RPL41 and SFRS4 (see Figure 2C) were used for normalization to assess the effects their choice for normalization has on the fold changes of differentially expressed genes during the course of HCV-induced HCC.

**Figure 3:**
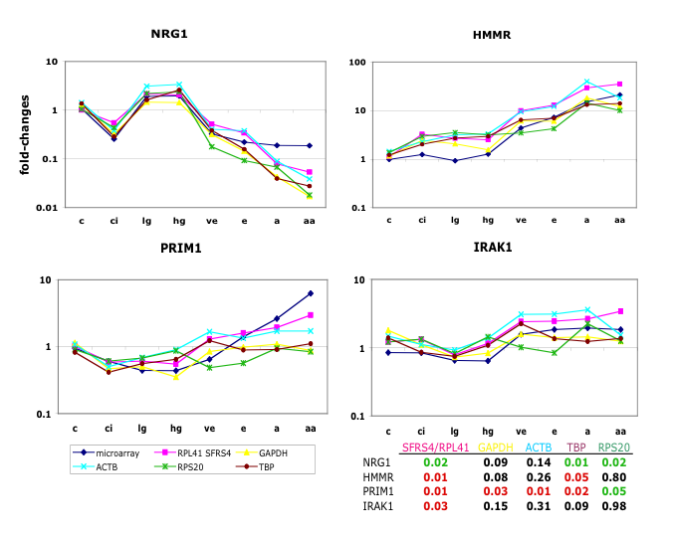
**Effects of reference genes used for normalization**: Relative expression of NRG1, HMMR, PRIM1, and IRAK1 for all stages of HCV-induced HCC. QPCR data were normalized to RPL41 and SFRS4 (shown in pink), to GAPDH (yellow), to ACTB (light blue), to RPS20 (green), and to TBP (brown). Microarray data are shown in dark blue. Fold-changes are indicated on the y-axis. Disease stages as in Figure 2. The table shows p-values for the change in gene expression from high-grade dysplasia to very early HCC for NRG1, HMMR, PRIM1, and IRAK1 (rows) when normalized to the genes indicated above (columns). Significant (p ≤ 0.5) up-regulation between these stages is indicated in red, down-regulation in green.

NRG1 was identified by microarray analysis to be decreased in cirrhosis, elevated in dysplasia, and again down-regulated during all four stages of HCC [4]. QPCR was performed on NRG1 to corroborate this expression pattern. Figure 3 shows the effects on relative NRG1 expression depending on which gene was used for normalization. All genes used for normalization were roughly able to confirm that pattern. However, the elevation of the resulting fold changes varied greatly. The up-regulation of NRG1 during dysplasia was much smaller, when GAPDH was used for normalization in comparison to the other reference genes. Similarly, the levels of down-regulation of NRG1 during the successive stages of HCC varied greatly dependent on the different reference genes.

HMMR was found via microarray technique to be not differentially expressed during the precancerous stages (cirrhosis, low- and high-grade dysplasia), followed by a significant increase for all HCC stages. QPCR corroboration, when normalized to RPL41 and SFRS4, GAPDH, ACTB, RPS20 or TBP revealed similar patterns with varying fold-changes (Figure 3). However, the increase in gene expression between high-grade dysplasia and very early HCC was very subtle when normalized to RPS20.

In the case of PRIM1, the choice of the normalization gene had dramatic effects on the relative gene expression. PRIM1 was found by microarray analysis to be down-regulated during cirrhosis, dysplasia and very early HCC, followed by increasing up-regulation in the successive stages of HCC. The most similar expression pattern resulted when the qPCR data were normalized to the combination of RPL41 and SFRS4 (Figure 3). The Student's t-test showed a significant increase between dysplasia and very early HCC (p = 0.011), confirming the significant increase found in the microarray analysis [4]. When ACTB was used for normalization the resulting fold changes were less evident but the tendency was similar. In contrast, normalization of PRIM1 using either GAPDH, RPS20, or TBP1 changed the expression pattern dramatically. For example, instead of being up-regulated, PRIM1 would be classified as down-regulated between high-grade dysplasia and very early HCC (p = 0.05, Figure 3).

A similar, albeit less dramatic effect is seen in the case of IRAK1. IRAK1 was slightly down-regulated during the precancerous stages of HCV-induced HCC, followed by small but significant up-regulation in HCC (Figure 3). Similar expression pattern were found, when the two genes, RPL41 and SFRS4 were used for normalization. Again, GAPDH, RPS20 and TBP changed even the tendency of the expression of IRAK1 in HCC.

These results clearly demonstrate the effects genes used for normalization have on the fold change of qPCR data and on the general direction (up or down) of differentially expressed genes.

## Discussion

The most commonly used reference genes for normalization of qPCR data are GAPDH and ACTB [17-24]. However, these genes can be significantly differentially expressed as shown in our study in HCV-induced HCC. GAPDH was strongly up-regulated in advanced and very advanced stages of HCC, in some samples up to 7-fold. ACTB was up-regulated two- to three-fold in many advanced and very advanced HCC samples. Also, ribosomal proteins should be considered individually, because many of them, e.g. RPS20 were differentially expressed during HCV-induced HCC [4], while RPL41 showed a relative stable expression throughout all stages of the disease.

It was reported that GAPDH and ACTB were also differentially expressed in other cancer types [8,14,33]. In bladder cancer, a study showed that GAPDH, G6PD and HMBS were significantly changed between malignant and nonmalignant tissues [25]. Similarly, in adenocarcinomas of the colon, the expression of RPLP0, RPS14 and GAPDH varied between primary tumors and corresponding resection margins [34]. Furthermore, in prostate cancer, ACTB, RPL13A and HMBS showed significant differences between cancer and noncancerous tissues [6]. Taken together, genes whose products have basic functions in cellular metabolisms are possibly differentially expressed between tumor and non-tumor tissues.

Normalization is used to adjust for experimental differences. This study presents an easy way to find appropriate candidates for normalization utilizing microarray data, also applicable to multiple comparisons. A pool of candidate genes can be found by selecting genes with low SD across all fold-changes and with similar signal intensity to the genes of interest (at least a present call). This identified the same best candidate, RPL41, as the procedure, in which differentially expressed genes were excluded.

We compared the qPCR data of six possible reference genes. The SD of the Ct values indicated that SFRS4 and RPL41 may be the best choice to be used for normalization. This was confirmed on the level of fold-changes, when we compared the CVs. Furthermore, the Student's t-test revealed that GAPDH, RPS20, TBP and ACTB were significantly regulated between certain stages of HCV-induced HCC. Consistent with these data, the geNorm-program also determined that SFRS4 and RPL41 were the most stable expressed genes. Using Normfinder [35], an additional computer program, aimed at identifying normalization genes, TBP was the best choice for normalization. However, we showed that TBP was significantly regulated between control and advanced HCC. In our situation, Normfinder was thus unable to identify the best normalization gene.

The effects of six genes used for normalization were compared on four differentially expressed genes: NRG1, HMMR, PRIM1, and IRAK1. In contrast to NRG1 and HMMR, where the resulting fold changes were over- and underestimated, depending on the gene used for normalization dramatic effects were found for the differentially expression of PRIM1 and IRAK1. Normalization using an inappropriate gene could lead to misinterpretation of the data, as it was shown for GAPDH, RPS20 or TBP in the context of HCV-induced HCC. Robust results were achieved by using two genes, RPL41 and SFRS4 in combination for normalization. Using at least two genes to normalize qPCR data has the advantages that they can compensate for slight differences in their expression. To profit most, these normalization genes should participate in different pathways.

This study, unlike many cancer studies, which compare tumor versus nontumor, comprised eight stages of HCV-induced HCC. Even though we included 72 tissue samples [4], each stage was only represented with seven to ten samples. This small sample size might be a limitation of the study design when performing statistical tests, such as t-tests between the stages. In order, to find the best normalization gene however, all samples were considered independent of their stage group.

Microarray data are known to be highly variable [36-41]. Due to its higher dynamic range qPCR, is thought to be more accurate and therefore is often used to corroborate microarray results [42,43]. Mostly, general direction (up- and down-regulation) and rank order of the fold-changes are similar, but the levels of the fold changes of microarray experiments differ compared to qPCR data [44-46] and show a marked tendency of being smaller [42,44,46]. This effect is more pronounced as the fold change ratio is very high [42].

This study shows the effects of reference genes used for normalization on qPCR data. The use of inappropriate genes for normalization can lead to an over- or under-estimation of the fold-changes or to misinterpretation of the results. The best results were achieved when the two genes RPL41 and SFRS4 were used for normalization.

## Conclusion

Many pathways are affected by cancer, as recently shown for HCC. Therefore, typical housekeeping genes or maintenance genes are likely to be differentially expressed during the course of the disease.

Appropriate genes for normalization should show a constant expression throughout all comparisons, they should be expressed in similar abundance as differentially expressed genes, and should not respond to the experimental treatment. From microarray experiments, genes, which display stable expression across all fold-changes are likely to be good candidates for normalization for qPCR. The utilization of at least two genes for normalization is highly recommended and will lead to the most reliable and accurate results.

In HCV-induced HCC the combination of RPL41 and SFRS4 were best to normalize qPCR data.

## Methods

### Tissue samples and microarray data

Tissue samples of this manuscript were described in [4]. To analyze hepatitis C virus (HCV) induced hepatocellular carcinoma (HCC) 72 tissue samples, including normal liver tissue (n = 10), cirrhotic liver tissue (n = 10), dysplastic nodules [low- (n = 10) and high-grade (n = 7)] and four successive stages of HCC [(from very early HCC to metastatic tumors with gross vascular invasion (n = 35)] were used to generate gene expression profiles by utilizing the human GeneChip whole genome array (U133 Plus 2.0 from Affymetrix). Data were normalized applying the GC Robust Multi-array Average (GC-RMA) algorithm and the baseline was calculated by the geometric mean using the data generated from 10 normal liver tissue samples (up- and down-regulation refers to the comparison with this baseline). Significant analysis of microarray (SAM) data was performed in GeneTraffic (Stratagene, La Jolla, CA). The microarray data are available at GEO (GSE 6764).

### RNA extraction

The tissue specimen were ground in liquid nitrogen and homogenized in Trizol (Invitrogen, Carlsbad, CA) using a polytron homogenizer. Total RNA was purified following the RNeasy Mini protocol (Qiagen, Valencia, CA), including a DNaseI digestion, to avoid contamination with genomic DNA. 28S/18S ratios measured with the Bioanalyzer (Agilent Technologies, Palo Alto, CA) had to be higher than 0.8 to be included into the study. Further quality criteria of the samples to be included into the study are described in detail elsewhere [4].

### QPCR

5 μg total, DNaseI treated RNA was reverse transcribed into cDNA using oligo dT and Superscript III (Invitrogen, Carlsbad, CA), followed by RNaseH digestion. The cDNA was diluted 1:100 and 5 μl were used as template in a 10 μl qPCR reaction. The qPCR assays were performed as described previously [43], using SYBR Green (Molecular Probes, Eugene, OR) and Platinum Taq (Invitrogen, Carlsbad, CA) on the ABI Prism 7900 (Applied Biosystems, Foster City, CA). Primers were designed using the Primer3 software [47]. The following primers were used: GAPDH (NM_002046) cga cca ctt tgt caa gct ca (sense) and agg ggt cta cat ggc aac tg (antisense); ACTB (NM_001101) gga ctt cga gca aga gat gg (sense) and agc act gtg ttg gcg tac ag (antisense); TBP1 (NM_003194) tat aat ccc aag cgg ttt gc (sense) and cac agc tcc cca cca tat tc (antisense), RPL41 (NM_021104) aag atg agg cag agg tcc aa (sense) and tcc aga atg tca cag gtc ca (antisense); SFRS4 (NM_005626) aaa agt cgg agc agg agt ca (sense) and ctc ttc ctg ccc ttc ctc tt (antisense); RPS20 (NM_001023) aac aag ccg caa cgt aaa at (sense) and gga aac gat ccc acg tct ta (antisense); PRIM1 (NM_000946) gcc ata cgc atc att gac ag (sense) and cca ccc ttt aca agg ctc aa (antisense); NRG1 (NM_004495) gcc tct gcc aat atc acc at (sense) and act ccc ctc cat tca cac ag (antisense); IRAK1 (NM_001569) gct ctt tgc cca tct ctt tg (sense) and gct acc acg cca ggc taa ta (antisense); and HMMR (NM_012485) tgc agc tca gga aca gct aa (sense) and caa gct gac agc gga gtt tt (antisense). Amplicon size and reaction specificity were confirmed by agarose gel electrophoresis and melting curve analysis. The PCR was performed after activation of the enzyme 95°C for 120s, for 40 cycles of: 95°C for 15s, 56°C for 15s and 72°C for 30s. The PCR reaction was followed by a dissociation curve 95°C for 15s, 60°C for 15s, 95°C for 15s (ramp 2%). All PCR reactions were performed in triplicate.

### Data analyses

The raw data were analyzed using SDS2.2 (Applied Biosystems, Foster City, CA) by subtraction the background and setting the threshold to obtain the Ct-value. PCR efficiencies (E) were calculated by using dilution series and the formula E = 10^(-1/slope)^-1. The efficiencies for the following PCRs were: GAPDH 0.89, ACTB 0.92, TBP 0.89, RPL41 0.78, SFRS4 0.94, RPS20 0.87, PRIM1 0.89, NRG1 0.97, IRAK1 0.96, and HMMR 0.89. All fold-changes were calculated based on these efficiencies. Further analyses were done in Excel: the median Ct was taken from triplicate reactions and compared to the median of all normal tissue samples, results are expressed as fold-changes. The qPCR reactions for RPL41, SFRS4, GAPDH, ACTB, RPS20, and TBP were done twice (in triplicates), independently to reduce the technical variation. The significance of differential expression was calculated by using the t-test in Excel. The geNorm analysis was performed as described in the manual [13]. Normfinder was used as in [48].

## Abbreviations

QPCR: quantitative real-time reverse transcriptase-PCR, HCC: hepatocellular carcinoma, HCV: hepatitis C virus, GAPDH: gene coding for glyceraldehyde-3- phosphate dehydrogenase, ACTB: gene coding for beta-actin, RPL41: gene coding for ribosomal protein large 41, RPS20: gene coding for ribosomal protein small 20, TBP: gene coding for TATA binding protein, SFRS4: gene coding for arginine/serine-rich splicing factor 4, SD: standard deviation, c: control, ci: cirrhosis, dn: dysplasia, lg: low-grade dysplasia, hg: high-grade dysplasia, ve: very early HCC, e: early HCC, a: advanced HCC, aa: very advanced HCC.

## Authors' contributions

EW conceived and designed the experiments, analyzed the data and wrote the manuscript. SW contributed to the discussion and commented the manuscript.

## References

[B1] Brumby AM, Richardson HE (2005). Using Drosophila melanogaster to map human cancer pathways. Nat Rev Cancer.

[B2] Kopper L, Timar J (2005). Genomics of prostate cancer: is there anything to "translate"?. Pathol Oncol Res.

[B3] Bianco R, Melisi D, Ciardiello F, Tortora G (2006). Key cancer cell signal transduction pathways as therapeutic targets. Eur J Cancer.

[B4] Wurmbach E, Chen YB, Khitrov G, Zhang W, Roayaie S, Schwartz M, Fiel I, Thung S, Mazzaferro V, Bruix J (2007). Genome-wide molecular profiles of HCV-induced dysplasia and hepatocellular carcinoma. Hepatology.

[B5] Revillion F, Pawlowski V, Hornez L, Peyrat JP (2000). Glyceraldehyde-3-phosphate dehydrogenase gene expression in human breast cancer. Eur J Cancer.

[B6] Ohl F, Jung M, Xu C, Stephan C, Rabien A, Burkhardt M, Nitsche A, Kristiansen G, Loening SA, Radonic A (2005). Gene expression studies in prostate cancer tissue: which reference gene should be selected for normalization?. J Mol Med.

[B7] Rubie C, Kempf K, Hans J, Su T, Tilton B, Georg T, Brittner B, Ludwig B, Schilling M (2005). Housekeeping gene variability in normal and cancerous colorectal, pancreatic, esophageal, gastric and hepatic tissues. Mol Cell Probes.

[B8] Khimani AH, Mhashilkar AM, Mikulskis A, O'Malley M, Liao J, Golenko EE, Mayer P, Chada S, Killian JB, Lott ST (2005). Housekeeping genes in cancer: normalization of array data. Biotechniques.

[B9] Thellin O, Zorzi W, Lakaye B, De Borman B, Coumans B, Hennen G, Grisar T, Igout A, Heinen E (1999). Housekeeping genes as internal standards: use and limits. J Biotechnol.

[B10] Warrington JA, Nair A, Mahadevappa M, Tsyganskaya M (2000). Comparison of human adult and fetal expression and identification of 535 housekeeping/maintenance genes. Physiol Genomics.

[B11] Hsiao LL, Dangond F, Yoshida T, Hong R, Jensen RV, Misra J, Dillon W, Lee KF, Clark KE, Haverty P (2001). A compendium of gene expression in normal human tissues. Physiol Genomics.

[B12] Butte AJ, Dzau VJ, Glueck SB (2001). Further defining housekeeping, or "maintenance," genes Focus on "A compendium of gene expression in normal human tissues". Physiol Genomics.

[B13] Vandesompele J, De Preter K, Pattyn F, Poppe B, Van Roy N, De Paepe A, Speleman F (2002). Accurate normalization of real-time quantitative RTPCR data by geometric averaging of multiple internal control genes. Genome Biol.

[B14] de Kok JB, Roelofs RW, Giesendorf BA, Pennings JL, Waas ET, Feuth T, Swinkels DW, Span PN (2005). Normalization of gene expression measurements in tumor tissues: comparison of 13 endogenous control genes. Lab Invest.

[B15] Kim KT, Baird K, Ahn JY, Meltzer P, Lilly M, Levis M, Small D (2005). Pim-1 is up-regulated by constitutively activated FLT3 and plays a role in FLT3-mediated cell survival. Blood.

[B16] Schmidt U, Fuessel S, Koch R, Baretton GB, Lohse A, Tomasetti S, Unversucht S, Froehner M, Wirth MP, Meye A (2006). Quantitative multi-gene expression profiling of primary prostate cancer. Prostate.

[B17] Heller RA, Schena M, Chai A, Shalon D, Bedilion T, Gilmore J, Woolley DE, Davis RW (1997). Discovery and analysis of inflammatory diseaserelated genes using cDNA microarrays. Proc Natl Acad Sci USA.

[B18] Chaib H, Cockrell EK, Rubin MA, Macoska JA (2001). Profiling and verification of gene expression patterns in normal and malignant human prostate tissues by cDNA microarray analysis. Neoplasia.

[B19] Schmidl M, Adam N, Surmann-Schmitt C, Hattori T, Stock M, Dietz U, Decrombrugghe B, Poschl E, von der Mark KC (2006). Twisted gastrulation modulates BMP- induced collagen II and X expression in chondrocytes in vitro and in vivo. J Biol Chem.

[B20] Thomas KE, Galligan CL, Deonarain Newman R, Fish EN, Vogel SN (2006). Contribution of interferon (IFN)-beta to the murine macrophage response to the TLR4 agonist, lipopolysaccharide. J Biol Chem.

[B21] Bianchini R, Nocentini G, Krausz LT, Fettucciari K, Coaccioli S, Ronchetti S, Riccardi C (2006). Modulation of pro- and anti-apoptotic molecules in double positive (CD4+CD8+) thymocytes following dexamethasone treatment. J Pharmacol Exp Ther.

[B22] Linsalata M, Giannini R, Notarnicola M, Cavallini A (2006). Peroxisome proliferator-activated receptor gamma and spermidine/spermine N1- acetyltransferase gene expressions are significantly correlated in human colorectal cancer. BMC Cancer.

[B23] Fok SY, Rubin JS, Pixley F, Condeelis J, Braet F, Soon LL (2006). Rapid chemokinetic movement and the invasive potential of lung cancer cells; a functional molecular study. BMC Cancer.

[B24] Fernandez-Cobo M, Holland JF, Pogo BG (2006). Transcription profiles of non-immortalized breast cancer cell lines. BMC Cancer.

[B25] Ohl F, Jung M, Radonic A, Sachs M, Loening SA, Jung K (2006). Identification and validation of suitable endogenous reference genes for gene expression studies of human bladder cancer. J Urol.

[B26] Huang R, Xing Z, Luan Z, Wu T, Wu X, Hu G (2003). A specific splicing variant of SVH, a novel human armadillo repeat protein, is up-regulated in hepatocellular carcinomas. Cancer Res.

[B27] Ormandy LA, Hillemann T, Wedemeyer H, Manns MP, Greten TF, Korangy F (2005). Increased populations of regulatory T cells in peripheral blood of patients with hepatocellular carcinoma. Cancer Res.

[B28] Qiu WH, Zhou BS, Chu PG, Chen WG, Chung C, Shih J, Hwu P, Yeh C, Lopez R, Yen Y (2005). Over-expression of fibroblast growth factor receptor 3 in human hepatocellular carcinoma. World J Gastroenterol.

[B29] Zhou J, Zhao LQ, Xiong MM, Wang XQ, Yang GR, Qiu ZL, Wu M, Liu ZH (2003). Gene expression profiles at different stages of human esophageal squamous cell carcinoma. World J Gastroenterol.

[B30] Jones J, Otu H, Spentzos D, Kolia S, Inan M, Beecken WD, Fellbaum C, Gu X, Joseph M, Pantuck AJ (2005). Gene signatures of progression and metastasis in renal cell cancer. Clin Cancer Res.

[B31] Nishida K, Mine S, Utsunomiya T, Inoue H, Okamoto M, Udagawa H, Hanai T, Mori M (2005). Global analysis of altered gene expressions during the process of esophageal squamous cell carcinogenesis in the rat: a study combined with a laser microdissection and a cDNA microarray. Cancer Res.

[B32] Saebo M, Skjelbred CF, Nexo BA, Wallin H, Hansteen IL, Vogel U, Kure EH (2006). Increased mRNA expression levels of ERCC1, OGG1 and RAI in colorectal adenomas and carcinomas. BMC Cancer.

[B33] Tricarico C, Pinzani P, Bianchi S, Paglierani M, Distante V, Pazzagli M, Bustin SA, Orlando C (2002). Quantitative real-time reverse transcription polymerase chain reaction: normalization to rRNA or single housekeeping genes is inappropriate for human tissue biopsies. Anal Biochem.

[B34] Dydensborg AB, Herring E, Auclair J, Tremblay E, Beaulieu JF (2006). Normalizing genes for quantitative RT-PCR in differentiating human intestinal epithelial cells and adenocarcinomas of the colon. Am J Physiol Gastrointest Liver Physiol.

[B35] Andersen CL, Jensen JL, Orntoft TF (2004). Normalization of real-time quantitative reverse transcription-PCR data: a model-based variance estimation approach to identify genes suited for normalization, applied to bladder and colon cancer data sets. Cancer Res.

[B36] Baldi P, Long AD (2001). A Bayesian framework for the analysis of microarray expression data: regularized t -test and statistical inferences of gene changes. Bioinformatics.

[B37] Lee ML, Kuo FC, Whitmore GA, Sklar J (2000). Importance of replication in microarray gene expression studies: statistical methods and evidence from repetitive cDNA hybridizations. Proc Natl Acad Sci USA.

[B38] Churchill GA (2002). Fundamentals of experimental design for cDNA microarrays. Nat Genet.

[B39] Love B, Rank DR, Penn SG, Jenkins DA, Thomas RS (2002). A conditional density error model for the statistical analysis of microarray data. Bioinformatics.

[B40] Novak JP, Sladek R, Hudson TJ (2002). Characterization of variability in large-scale gene expression data: implications for study design. Genomics.

[B41] Chen JJ, Delongchamp RR, Tsai CA, Hsueh HM, Sistare F, Thompson KL, Desai VG, Fuscoe JC (2004). Analysis of variance components in gene expression data. Bioinformatics.

[B42] Yuen T, Wurmbach E, Pfeffer RL, Ebersole BJ, Sealfon SC (2002). Accuracy and calibration of commercial oligonucleotide and custom cDNA microarrays. Nucleic Acids Res.

[B43] Wurmbach E, Yuen T, Sealfon SC (2003). Focused microarray analysis. Methods.

[B44] Wurmbach E, Yuen T, Ebersole BJ, Sealfon SC (2001). Gonadotropinreleasing hormone receptor-coupled gene network organization. J Biol Chem.

[B45] Wu CG, Budhu A, Chen S, Zhou X, Popescu NC, Valerie K, Wang XW (2006). Effect of hepatitis C virus core protein on the molecular profiling of human B lymphocytes. Mol Med.

[B46] Li RW, Waldbieser GC (2006). Production and utilization of a high-density oligonucleotide microarray in channel catfish, Ictalurus punctatus. BMC Genomics.

[B47] http://frodo.wi.mit.edu/cgi-bin/primer3/primer3_www.cgi.

[B48] http://www.mdl.dk/publicationsnormfinder.htm.

